# A feasibility study of computed tomography muscle phenotyping for physiotherapy selection in peripheral arterial disease

**DOI:** 10.1016/j.jvscit.2026.102273

**Published:** 2026-04-29

**Authors:** Matej Pekar, Barbora Ryskova, Lubomir Blaha, Jana Dostalova, Marek Kantor, Otakar Jiravsky

**Affiliations:** a Complex Cardiovascular Centre, Hospital AGEL Trinec-Podlesi, Trinec, Czech Republic; b Department of Physiology, Faculty of Medicine, Masaryk University, Brno, Czech Republic

**Keywords:** Peripheral arterial disease, Muscle quality, Body composition, Computed tomography, Sarcopenic obesity, Physiotherapy, Patient selection

## Abstract

**Objective:**

Patients with peripheral arterial disease commonly undergo computed tomography (CT) angiography for anatomical assessment, yet body composition data from these scans remain unexploited for treatment planning. We evaluated the feasibility of extracting metabolic phenotype information from routine CT imaging and explored whether baseline body composition parameters might relate to physiotherapy outcomes in this hypothesis-generating pilot investigation.

**Methods:**

This prospective feasibility study enrolled 10 patients with symptomatic peripheral arterial disease (Fontaine stages IIa-IIb); eight completed a 3-month supervised physiotherapy program consisting of progressive treadmill walking to moderate claudication and resistance training. We performed CT body composition analysis at the L3 vertebral level at baseline and follow-up, measuring the skeletal muscle index (SMI), muscle density in Hounsfield units (HU), visceral adipose tissue area, and subcutaneous adipose tissue area using automated segmentation. Functional assessments included the chair stand test, handgrip strength, and 2-minute walk test. The primary outcome was treatment success (continued conservative management) vs failure (revascularization required). We explored correlations between baseline body composition parameters and functional performance using Spearman rank correlation.

**Results:**

CT body composition analysis proved technically feasible in all patients, requiring minimal additional processing time. Five patients (62.5%) achieved treatment success. Baseline muscle density showed correlation with chair stand performance (ρ = 0.829; *P* = .021), whereas the SMI showed no functional relationships. Visceral adipose tissue was associated in five patients with walking distance (ρ= −0.900; *P* = .037) and handgrip strength (ρ = −0.775; *P* = .041). Patients achieving treatment success had a lower body mass index (24.9 kg/m^2^ vs 29.6 kg/m^2^) and higher muscle density (41.1 HU vs 35.9 HU). Paradoxically, patients requiring revascularization had higher SMI (59.7 cm^2^/m^2^ vs 48.6 cm^2^/m^2^) but lower muscle quality. Body composition changes during the intervention showed no relationship to outcomes.

**Conclusions:**

This feasibility study demonstrates that metabolic phenotyping using existing CT imaging is technically viable in patients with peripheral arterial disease undergoing physiotherapy evaluation. Exploratory observations suggest testable hypotheses: that muscle quality may prove more functionally relevant than muscle quantity, that visceral adiposity may associate with impaired rehabilitation capacity, and that myosteatotic obesity could emerge as a high-risk phenotype. These preliminary findings support the rationale for adequately powered prospective studies to validate whether CT-based metabolic phenotyping could inform patient selection for the conservative management of claudication.

**Article Highlights**

• **Type of Research:** Prospective pilot feasibility study
• **Key Findings:** Automated computed tomography body composition analysis at the L3 vertebral level is technically feasible within routine clinical workflows for peripheral arterial disease patients with no additional radiation exposure or cost. Baseline muscle density (a marker of muscle quality) correlated strongly with lower extremity functional performance (ρ = +0.829), whereas skeletal muscle index (muscle quantity) showed no functional relationships. Visceral adipose tissue area demonstrated the strongest associations with functional capacity, correlating inversely with walking distance (ρ = −0.900) and handgrip strength (ρ = −0.775). A paradoxical myosteatotic obesity phenotype was identified: patients requiring revascularization had greater muscle mass but lower muscle density and higher body mass index than those who succeeded with conservative management.
• **Take Home Message:** Muscle quality and visceral adiposity—not muscle quantity—appear to be the dominant metabolic determinants of physiotherapy response in peripheral arterial disease. Computed tomography-based metabolic phenotyping, extractable from imaging already obtained for anatomical vascular assessment, represents a practical, zero-burden tool that warrants validation in adequately powered prospective studies for physiotherapy patient selection in claudication management.

Supervised exercise therapy represents the cornerstone of conservative management for intermittent claudication, universally recommended as first-line treatment before revascularization is considered.^1^, ^2^, ^3^ This standardized approach to all appropriate candidates overlooks a fundamental clinical reality: physiotherapy outcomes vary dramatically among patients with similar anatomical disease severity and claudication symptoms. This variability represents both a clinical challenge and a resource allocation problem, because intensive physiotherapy programs demand substantial health care investment in supervised sessions, home exercise protocols, and physiotherapy time without reliable methods to predict which patients will benefit.^4^^,^^5^

Current patient selection mechanisms for conservative claudication management rely primarily on anatomical suitability and symptom severity, with limited tools to assess individual metabolic capacity for rehabilitation. Traditional prognostic factors, including the ankle-brachial index and baseline claudication distance, show inconsistent relationships with physiotherapy response, leaving clinicians without objective criteria for treatment selection.^6^ The absence of validated selection tools means that patients are often committed to lengthy rehabilitation programs without prior assessment of their metabolic reserve to respond to exercise training.^7^

Body composition parameters, including muscle quality, muscle quantity, and visceral adiposity, can be extracted from standard cross-sectional imaging at the third lumbar vertebral level using automated segmentation,^8^ yet these data are rarely considered when selecting patients for conservative therapy. The existing literature on body composition in peripheral arterial disease has focused predominantly on muscle quantity, particularly sarcopenia defined as low muscle mass,^9^^,^^10^ whereas muscle quality, which reflects metabolic health and intramuscular lipid infiltration, has received comparatively little attention.^10^ Similarly, the impact of visceral adiposity on functional rehabilitation capacity in claudicants remains unexplored, despite extensive evidence linking visceral fat to metabolic dysfunction and impaired exercise capacity in other populations.^11^

Automated artificial intelligence-based segmentation now makes comprehensive body composition analysis feasible within clinical workflows, requiring only seconds of processing time beyond standard imaging interpretation.^12^ Muscle density reflects intramuscular lipid infiltration and metabolic health,^13^ potentially providing superior functional prediction compared with muscle mass alone.^9^^,^^10^^,^^14^ Furthermore, the simultaneous assessment of visceral and subcutaneous adipose tissue compartments may identify the sarcopenic obesity phenotype, wherein abundant but metabolically dysfunctional muscle coexists with high visceral adiposity.^15^ By extracting this comprehensive metabolic phenotype from computed tomography (CT) scans already obtained for anatomical vascular assessment, clinicians could potentially identify baseline metabolic reserve before committing patients to resource-intensive rehabilitation programs with zero additional radiation exposure, cost, or patient burden.

This prospective pilot investigation addresses two fundamental questions: whether CT muscle phenotyping is technically achievable in real-world clinical practice, and whether baseline metabolic parameters show a sufficient relationship with physiotherapy outcomes to warrant validation in larger cohorts. Explicitly positioned as hypothesis generating rather than confirmatory, the primary aim of this work was to demonstrate the technical feasibility of automated body composition analysis within standard clinical workflows; secondary aims explored relationships between baseline muscle quality, muscle quantity, visceral adiposity, and physiotherapy response, and characterized metabolic phenotypes associated with treatment success vs revascularization.

## Methods

### Study design and setting

This prospective pilot case series enrolled 10 patients with symptomatic peripheral arterial disease at a tertiary Complex Cardiovascular Centre in the Czech Republic, between February 2023 and September 2025, with eight completing the 3-month physiotherapy protocol. The study received approval from the institutional Ethics Committee (approval number EK 314/22), and all participants provided written informed consent. This investigation assessed the feasibility of extracting metabolic phenotype information from routine clinical imaging and explored the relationships between baseline body composition parameters and physiotherapy outcomes.

### Patient population

Eligible patients presented with symptomatic peripheral arterial disease classified as Fontaine stages IIa or IIb (Rutherford categories 1-3), with vascular anatomy amenable to revascularization but appropriate for initial conservative management. The cohort represented a real-world claudication population with substantial comorbidity burden, including universal prevalence of ischemic heart disease, pulmonary disease in 50%, and chronic renal insufficiency in 37%. The mean patient age was 62 ± 8 years (range, 49-73 years), 63% were male, and their body mass index (BMI) averaged 26 ± 6 kg/m^2^. Obesity, defined as a BMI of ≥30 kg/m^2^, was present in 38% of patients. Baseline claudication distance averaged 104 m (range, 20-300 m). This comorbidity profile reflects typical clinical practice populations, strengthening the generalizability of our observations.

### CT body composition analysis

CT scans obtained for routine anatomical vascular assessment were analyzed for body composition using automated artificial intelligence-based segmentation by AutoMATiCA, requiring minimal additional processing time beyond standard clinical imaging workflows. This approach represents the core innovation, repurposing existing diagnostic imaging to extract prognostic metabolic information without additional radiation exposure, cost, or patient burden. Scans were performed at baseline and the 3-month follow-up, with analysis conducted at the third lumbar vertebra level, a validated anatomical landmark correlating with whole body composition.^8^ Automated segmentation identified tissue compartments using established Hounsfield unit thresholds: skeletal muscle, −29 to +150 Hounsfield units (HU); visceral adipose tissue, −150 to −50 HU; and subcutaneous adipose tissue, −190 to −30 HU. The skeletal muscle index (SMI) was calculated by dividing muscle cross-sectional area by height squared. Muscle density, reflecting intramuscular lipid infiltration, was quantified as the mean HU value across skeletal muscle pixels. Visceral and subcutaneous adipose tissue areas were measured in square centimeters, and whole body fat mass percentage was estimated using validated single-slice regression equations. This comprehensive assessment provided quantitative measures of muscle and fat mass alongside qualitative indicators of tissue metabolic health.

### Functional performance testing

Standardized assessments were administered at baseline and the 3-month follow-up. The chair stand test measured lower extremity strength by counting sit-to-stand repetitions in 30 seconds. Handgrip strength was assessed bilaterally using a calibrated dynamometer, with maximum force across three trials per hand recorded. The 2-minute walk test quantified functional capacity by measuring distance covered during continuous self-paced walking. Baseline claudication distance was assessed during treadmill walking.

### Physiotherapy intervention

Participants underwent a structured three-month supervised program (mean duration, 86 days; range, 64-143 days) comprising six in-clinic sessions (days 1, 14, 30, 44, 67, and 90) lasting 45-60 minutes each. Sessions included breathing exercises, progressive resistance training with elastic bands, supervised treadmill walking to moderate claudication pain (Borg scale of 12-13) with rest intervals, and aerobic conditioning using stationary bicycles, elliptical machines, and rowing machines. Between sessions, participants followed a progressive home program beginning with 15-minute walks three times weekly and resistance exercises (eight repetitions, two sets), advancing to 30-minute walks and intensified resistance training (12 repetitions, 5 sets, approximately 70% one-repetition maximum). This blended approach reflects pragmatic contemporary physiotherapy delivery.

### Outcome definitions

The primary outcome distinguished treatment success (continued conservative management) from failure (revascularization required due to clinical deterioration or patient preference after inadequate symptom relief). Revascularization decisions involved clinical judgment incorporating symptom severity, functional limitation, and patient preferences. Secondary outcomes included changes in functional performance and body composition parameters.

### Statistical analyses

Given the pilot nature and small sample size, analyses were exploratory and hypothesis generating rather than confirmatory. Nonparametric methods were used throughout. Baseline characteristics were compared between success and failure groups using the Mann-Whitney *U* test for continuous variables and Fisher's exact test for categorical variables. Relationships between body composition and functional performance were examined using Spearman's rank correlation. Pre-post changes were assessed using Wilcoxon signed-rank test. Statistical significance was defined as a *P* value of <.05, without correction for multiple comparisons, given the exploratory objectives. Emphasis was placed on effect sizes and clinical meaningfulness alongside statistical significance. Missing data were handled using complete case analysis with pairwise deletion without imputation.

## Results

Ten patients with symptomatic peripheral arterial disease were enrolled between February 2023 and September 2025, of whom 8 completed the 3-month physiotherapy protocol, representing a 20% dropout rate. Complete baseline and follow-up CT body composition data were available for six patients; baseline CT analysis was successfully performed in seven. Functional performance assessments at 3 months were completed in seven patients for the chair stand test and handgrip strength, and six patients for the 2-minute walk test. Body composition analysis using automated segmentation at the third lumbar vertebral level proved technically feasible in all patients, requiring minimal additional processing time beyond standard clinical imaging workflows.

The cohort reflected a real-world claudication population with substantial comorbidity burden. The mean patient age was 62 ± 8 years (range, 49-73 years), 63% were male, and their BMI averaged 26 ± 6 kg/m^2^. All patients had concomitant ischemic heart disease, 50% had pulmonary disease, and 37% had chronic renal insufficiency. The majority presented with advanced claudication, with 87.5% classified as Fontaine stage IIb and a mean claudication distance of 104 ± 93 m (range, 20-300 m). Obesity was present in 37% of patients at baseline. Baseline characteristics are detailed in Table I.

**Table I:** Patient demographics, comorbidities, and disease severity by treatment outcome

Variable	Overall (n = 8)	Treatment success (n = 5)	Treatment failure (n = 3)
Demographics			
Age, years	62.0 ± 8.4	63.2 ± 7.0	59.0 ± 14.1
Male sex	5 (62.5%)	3 (60.0%)	2 (66.7%)
Height, cm	171.6 ± 9.0	—	—
Weight, kg	76.1 ± 10.1	—	—
BMI, kg/m^2^	26.2 ± 5.5	24.9 ± 4.4	29.6 ± 8.5
BMI ≥30 kg/m^2^	3 (37.5%)	0 (0%)	3 (100%)
Comorbidities			
Ischemic heart disease	8 (100%)	5 (100%)	3 (100%)
Pulmonary disease	4 (50.0%)	—	—
Chronic renal insufficiency	3 (37.5%)	—	—
Current/former smoking	2 (25.0%)	2 (40.0%)	0 (0%)
Diabetes mellitus	1 (12.5%)	0 (0%)	1 (33.3%)
Hypertension	0 (0%)	0 (0%)	0 (0%)
Vascular disease severity			
Claudication distance, m	104.3 ± 92.7	120.0 ± 105.8	65.0 ± 49.5
Fontaine stage IIa	1 (12.5%)	—	—
Fontaine stage IIb	7 (87.5%)	—	—
Baseline functional performance			
Chair stand test, repetitions	18.4 ± 7.2	18.6 ± 8.3	18.0 ± 5.7
Handgrip strength, kg	33.0 ± 11.9	33.7 ± 12.4	31.4 ± 15.1
2-Minute walk test, m (n = 6)	114.7 ± 39.8	116.3 ± 22.6	108.0 ± 82.0

*BMI*, Body mass index.

Data are presented as mean ± standard deviation or number (%).

*Dashes* indicate data not stratified by outcome for that variable.

Baseline body composition analysis at the third lumbar vertebral level revealed substantial interindividual variability in metabolic phenotypes despite similar claudication severity. The mean SMI was 51.8 ± 13.9 cm^2^/m^2^ (n = 7), muscle density averaged 39.6 ± 11.7 HU, visceral adipose tissue (VAT) area measured 154.3 ± 94.1 cm^2^, and subcutaneous adipose tissue measured 122.0 ± 71.5 cm^2^. The large standard deviations, particularly for adipose tissue compartments, highlights the marked metabolic heterogeneity within this small cohort (Table II).

**Table II:** Baseline computed tomography (CT) body composition parameters by treatment outcome

Parameter	Overall (n = 7)	Treatment success (n = 5)	Treatment failure (n = 2)
Muscle parameters			
SMI, cm^2^/m^2^	51.8 ± 13.9	48.6 ± 14.6	59.7 ± 11.6
Muscle area, cm^2^	150.1 ± 31.2	144.2 ± 35.8	164.8 ± 9.9
Muscle density, HU	39.6 ± 11.7	41.1 ± 10.7	35.9 ± 18.2
Adipose tissue parameters			
VAT, cm^2^	154.3 ± 94.1	164.7 ± 102.5	128.3 ± 95.9
Subcutaneous adipose tissue, cm^2^	122.0 ± 71.5	111.2 ± 71.9	149.0 ± 89.5
Whole body fat mass, %	30.2 ± 6.5	30.9 ± 7.2	28.5 ± 5.7

*HU*, Hounsfield units; *SMI*, skeletal muscle index; *VAT*, visceral adipose tissue.

Data are mean ± standard deviation.

CT analysis performed at the L3 vertebral level using automated artificial intelligence-based segmentation. Body composition available in seven of eight patients at baseline (one patient was missing a baseline CT scan); treatment outcome body composition available in five success and two failure patients only.

Following the 3-month intervention, treatment success was achieved in five of the eight patients (62.5%) who maintained conservative management; three patients (37.5%) required revascularization. Functional performance changes from baseline to follow-up demonstrated modest improvements with high individual variability. The 2-minute walk test distance increased by 19 ± 47 m (n = 6), the chair stand test performance declined slightly by 1.0 ± 3.3 repetitions (n = 7), and handgrip strength improved minimally by 1.4 ± 8.0 kg (n = 7). Body composition changes over the intervention period were similarly modest. Among six patients with complete paired data, muscle area decreased by 1.5 ± 28.5 cm^2^, The SMI declined by 0.7 ± 9.5 cm^2^/m^2^, and muscle density showed a progressive decline of 5.8 ± 8.2 HU. Visceral and subcutaneous adipose tissue areas remained essentially unchanged, with mean changes of −4.9 and +0.1 cm^2^, respectively (Table III).

**Table III:** Functional performance and computed tomography (CT) body composition parameters at baseline and the 3-month follow-up

Parameter	Baseline	3-Month follow-up	Change
Functional performance			
Chair stand test, repetitions (n = 7)	18.4 ± 7.2	17.4 ± 6.6	−1.0 ± 3.3
Handgrip strength, kg (n = 7)	33.0 ± 11.9	34.4 ± 13.1	+1.4 ± 8.0
2-Minute walk test, m (n = 6)	114.7 ± 39.8	133.8 ± 55.9	+19.2 ± 47.3
CT body composition (n = 6)			
SMI, cm^2^/m^2^	51.0 ± 15.2	50.3 ± 12.7	−0.7 ± 9.5
Muscle area, cm^2^	148.5 ± 34.3	147.0 ± 24.7	−1.5 ± 28.5
Muscle density, HU	39.0 ± 12.6	33.2 ± 10.6	−5.8 ± 8.2
VAT, cm^2^	147.2 ± 107.4	142.3 ± 87.4	−4.9 ± 43.5
Subcutaneous adipose tissue, cm^2^	122.9 ± 78.5	123.0 ± 83.1	+0.1 ± 65.8
Whole body fat mass, %	29.5 ± 6.6	29.1 ± 5.9	−0.4 ± 3.4

*HU*, Hounsfield units; *SMI*, skeletal muscle index; *VAT*, visceral adipose tissue.

Data are mean ± standard deviation.

Complete paired functional data are available for seven patients for the chair stand test and handgrip strength and six patients for the 2-minute walk test. Complete paired CT data are available in six patients. Positive change values indicate improvement; negative values indicate decline.

**Fig 1:**
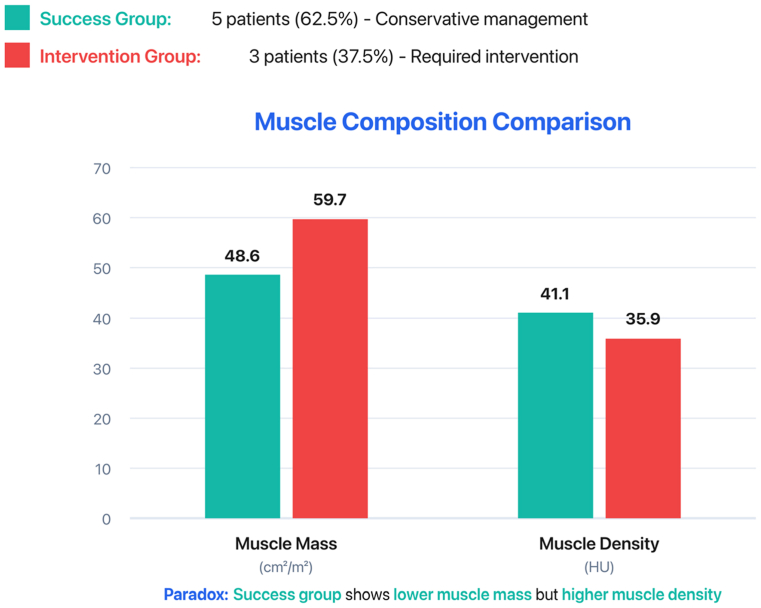
Baseline muscle composition parameters by treatment outcome. Bar graphs comparing skeletal muscle index (SMI) and muscle density between patients achieving treatment success (continued conservative management) and treatment failure (revascularization required). Patients requiring revascularization demonstrated paradoxically higher muscle mass but lower muscle density compared with those who maintained conservative management, consistent with a sarcopenic obesity phenotype. *HU*, Hounsfield units.

A paradoxical pattern emerged when comparing baseline characteristics between treatment outcome groups. Patients achieving treatment success demonstrated a lower SMI (48.6 vs 59.7 cm^2^/m^2^; n = 5 and 2, respectively), but greater muscle density (41.1 HU vs 35.9 HU) (Fig 1). BMI was lower in the success group (24.9 kg/m^2^ vs 29.6 kg/m^2^; n = 5 and 3, respectively), and obesity prevalence differed markedly, with zero of five success patients meeting obesity criteria compared with all three failure patients. This quantity-quality paradox suggested that patients requiring revascularization possessed greater muscle mass but inferior muscle quality, consistent with a myosteatotic obesity phenotype (Table II).

**Fig 2:**
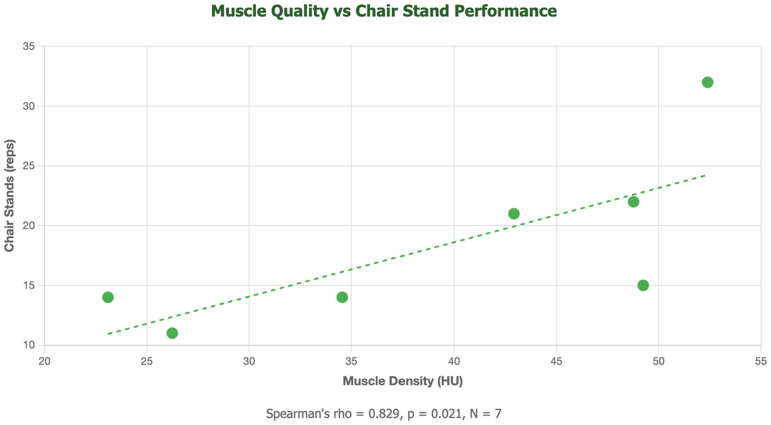
Baseline muscle density correlates with lower extremity functional performance. Scatter plot illustrating the relationship between baseline muscle density and chair stand test performance in patients with peripheral arterial disease (n = 7). Each data point represents one patient. The *dashed line* represents linear regression. Greater muscle density, reflecting preserved tissue quality and reduced intramuscular lipid infiltration, was associated with greater lower extremity functional performance (Spearman's ρ = +0.829; *P* = .021). *HU*, Hounsfield units.

Baseline muscle density demonstrated a strong positive correlation with chair stand test performance in this small cohort (Spearman's ρ = +0.829; *P* = .021; n = 7) (Fig 2), whereas the SMI showed no significant relationship with any functional parameter. Greater muscle density, reflecting preserved tissue quality and reduced intramuscular lipid infiltration, was associated with better lower extremity functional performance. In contrast, muscle quantity as measured by the SMI or absolute muscle area failed to predict functional capacity, suggesting that qualitative muscle characteristics may prove more functionally relevant than quantitative measures in this patient population, although these preliminary observations require validation in larger cohorts.

**Fig 3:**
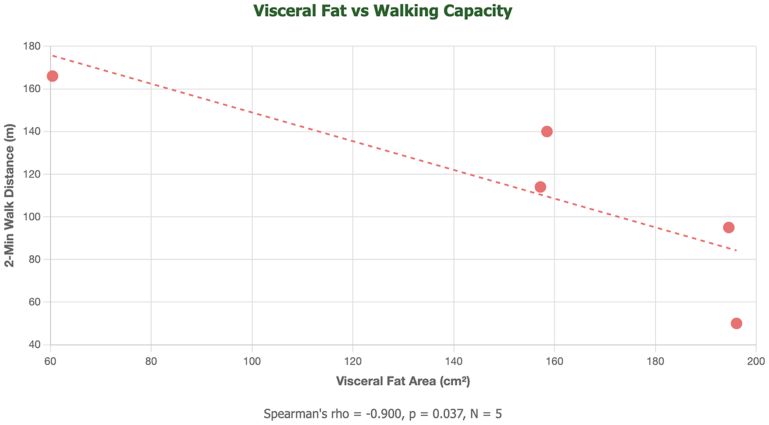
Visceral adiposity associates inversely with walking capacity. Scatter plot illustrating the relationship between baseline visceral adipose tissue (VAT) area and 2-minute walk test distance in patients with available walk test data (n = 5). Each data point represents one patient. The *dashed line* represents linear regression. Greater VAT area was strongly associated with reduced functional walking capacity (Spearman's ρ = −0.900; *P* = .037).

**Fig 4:**
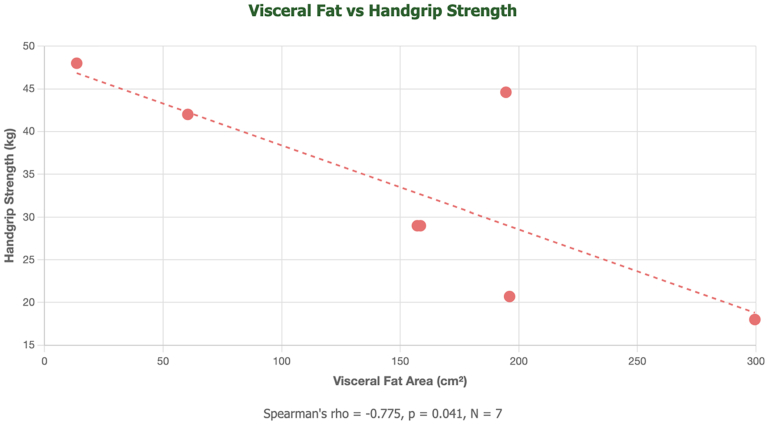
Visceral adiposity associates inversely with overall muscle strength. Scatter plot illustrating the relationship between baseline visceral adipose tissue (VAT) area and handgrip strength in patients with peripheral arterial disease (n = 7). Each data point represents one patient. The *dashed line* represents linear regression. Greater visceral adipose tissue (VAT) area was associated with reduced grip strength, suggesting that visceral adiposity impairs systemic functional reserve, independent of muscle mass (Spearman's ρ = −0.775; *P* = .041).

VAT area demonstrated the strongest associations with functional capacity observed in this study. A very strong negative correlation emerged between VAT and the 2-minute walk test distance (ρ = −0.900; *P* = .037; n = 5) (Fig 3), representing the most robust correlation identified in this exploratory analysis. VAT also showed a significant negative correlation with handgrip strength (ρ = −0.775; *P* = .041; n = 7) (Fig 4). Paradoxically, VAT area correlated positively with the SMI (ρ = +0.821; *P* = .023; n = 7), suggesting a myosteatotic obesity signature wherein increased muscle mass coexisted with high visceral adiposity and poor functional performance. These significant correlations are summarized in Table IV.

**Table IV:** Significant Spearman rank correlations between baseline body composition and functional performance parameters

Body composition parameter	Functional parameter	Spearman's ρ	*P* value	No.
Muscle density (HU)	Chair stand test	+0.829	0.021	7
VAT (cm^2^)	2-Minute walk test	−0.900	0.037	5
VAT (cm^2^)	Handgrip strength	−0.775	0.041	7
VAT (cm^2^)	SMI	+0.821	0.023	7

*HU*, Hounsfield units; *SMI*, skeletal muscle index; *VAT*, visceral adipose tissue.

All correlations are Spearman's rank correlation coefficients. Statistical significance threshold *P* < .05; no correction for multiple comparisons applied (exploratory pilot study). No nonsignificant correlations are shown.

Changes in body composition parameters during the intervention showed no significant correlation with changes in functional performance or treatment outcome in this small cohort. Individual cases illustrated this disconnect between training-induced adaptations and clinical outcomes. One patient experienced the largest muscle area loss (32 cm^2^), yet achieved treatment success and continued conservative management. Conversely, another patient gained muscle mass (10 cm^2^) and demonstrated a high baseline SMI (68.0 cm^2^/m^2^), but possessed extremely low muscle density (23.1 HU) and ultimately required revascularization. These observations suggest that pre-existing metabolic capacity, rather than plasticity in response to exercise training, may distinguish responders from nonresponders to physiotherapy in this feasibility investigation.

## Discussion

This feasibility study demonstrates that comprehensive metabolic phenotyping using CT body composition analysis is technically viable and clinically practical for patients with peripheral arterial disease undergoing physiotherapy evaluation. Because most patients with symptomatic peripheral arterial disease undergo CT angiography (CTA) to define anatomical targets for revascularization, the metabolic phenotype information inherent in these same images can be extracted using automated segmentation requiring only seconds of additional processing time.^12^^,^^16^^,^^17^ This approach provides clinicians with an objective baseline metabolic assessment before committing patients to resource-intensive 3-month rehabilitation programs, with zero additional radiation exposure, cost, or patient burden.^12^^,^^18^ In this small pilot cohort, we observed patterns that, although requiring cautious interpretation, suggest testable hypotheses worthy of validation in larger prospective studies.

The paradoxical relationship between muscle quantity and quality emerged as perhaps the most striking observation in this exploratory analysis. Baseline muscle density demonstrated a strong correlation with chair stand performance, whereas the SMI showed no significant relationship with any functional parameter. Patients who ultimately required revascularization possessed greater muscle mass than those who achieved treatment success, yet exhibited inferior muscle quality as reflected by lower tissue density. It must be emphasized, however, that baseline CT body composition data were available in only two of three failure patients, rendering this subgroup observation particularly fragile. As an alternative explanation, patients with greater muscle mass may have been more physically active before disease onset, and their physiotherapy failure may reflect advanced local ischemia rather than systemic metabolic insufficiency. Muscle density measured by HU reflects intramuscular lipid infiltration, with a lower density indicating metabolic dysfunction, impaired mitochondrial function, and decreased oxidative capacity.^13^^,^^19^ High-density muscle suggests preserved oxidative metabolism, which is essential for endurance exercise performance, particularly the sustained walking to moderate claudication pain that forms the foundation of physiotherapy protocols.^19^^,^^20^ Although these mechanisms are well characterized in oncology,^21^, ^22^, ^23^ heart failure,^24^ and aging populations,^19^^,^^20^ this work represents their application to peripheral arterial disease rehabilitation selection. The implication is profound: abundant muscle tissue without metabolic quality represents dysfunctional capacity unlikely to adapt effectively to aerobic training stimulus within standard 3-month timeframes.

VAT area demonstrated the strongest association with functional capacity observed in this study, with a very strong negative correlation with the 2-minute walk test distance, representing the most robust relationship identified. This finding appeared to be consistent across both local functional measures such as walking capacity and systemic measures, including handgrip strength, suggesting that visceral fat accumulation may represent a primary metabolic barrier to rehabilitation. The mechanisms underlying this association are biologically plausible and well documented in the cardiovascular literature.^11^^,^^25^ VAT functions as a metabolically active endocrine organ producing proinflammatory adipokines that drive systemic inflammation, impair endothelial function, and decrease insulin sensitivity.^26^^,^^27^ In patients with peripheral arterial disease specifically, this metabolic dysfunction compounds existing vascular limitations by creating a hostile environment for exercise adaptation.^28^^,^^29^ The observed positive correlation between VAT and SMI further suggested a myosteatotic obesity signature wherein increased muscle mass coexists with high visceral adiposity and poor functional performance.^15^^,^^30^ This combined phenotype may represent a particularly high-risk group for conservative management, yet these patients might appear deceptively robust on traditional assessment focused on muscle mass alone. The clinical implication is that visceral fat represents a modifiable metabolic barrier, although one that is unlikely to change meaningfully within the standard 3-month physiotherapy timeframe.^31^^,^^32^ Patients with substantial visceral adiposity may require pre-rehabilitation metabolic optimization through targeted weight loss and glycemic control,^32^, ^33^, ^34^ or alternatively, earlier consideration of revascularization rather than prolonged conservative trials.^1^^,^^7^

The disconnect between body composition changes during the intervention and clinical outcomes provides important insight into physiotherapy response determinants. Changes in muscle mass, muscle quality, and adipose tissue compartments showed no significant correlation with functional improvements or treatment success, and individual cases illustrated this paradox strikingly: the patient with the largest muscle area loss achieved treatment success, whereas another who gained muscle mass possessed an extremely low baseline muscle density and ultimately required revascularization. These observations suggest that pre-existing metabolic capacity, rather than plasticity in response to exercise training, distinguishes responders from nonresponders.^31^ The minimal body composition remodeling observed over 3 months^31^^,^^32^ implies that baseline metabolic phenotype determines treatment trajectory, challenging the assumption that all patients with claudication can build functional capacity through standardized exercise protocols within conventional timeframes.^7^^,^^35^ Such patients may benefit from metabolic optimization before physiotherapy initiation^33^^,^^34^ or earlier revascularization consideration.^1^^,^^7^

The potential clinical application of CT-based metabolic phenotyping centers on pre-rehabilitation risk stratification using imaging already obtained for anatomical planning. In practice, this approach could identify patients with favorable metabolic phenotypes—characterized by low visceral adiposity, high muscle density, and absence of obesity—allowing clinicians to proceed confidently with standard physiotherapy, knowing these patients possess the metabolic reserve to respond. Conversely, the identification of high-risk phenotypes characterized by myosteatotic obesity could prompt modified treatment approaches, including metabolic optimization before physiotherapy, intensified or prolonged rehabilitation, or earlier revascularization consideration.^1^^,^^15^ This approach represents a shift from universal exercise prescription toward phenotype-guided treatment selection based on objective, reproducible biomarkers.^1^^,^^7^^,^^31^ Importantly, this approach requires no additional cost, radiation exposure, or patient burden because the requisite data are generated during standard diagnostic imaging workflows.^12^^,^^17^

The applicability of CT-based metabolic phenotyping depends on CTA being available before the physiotherapy decision, which varies across centers. In many community settings, CTA is reserved for revascularization planning, limiting its utility for conservative management selection. In tertiary centers and systems following the 2024 European Society of Cardiology Guidelines, CTA is often obtained earlier, creating a natural opportunity to extract metabolic phenotype data from the same dataset. Where this is not routine, future work should explore whether opportunistic body composition analysis or ultrasound-derived visceral adiposity estimates could provide equivalent information without added patient burden.

The existing literature provides limited guidance on body composition predictors of physiotherapy response in peripheral arterial disease, with most prior work focusing on sarcopenia as a mortality or amputation risk factor rather than a determinant of functional rehabilitation success.^9^^,^^36^, ^37^, ^38^ Visceral adiposity remains underexplored in vascular disease populations despite extensive evidence linking visceral fat to metabolic dysfunction and impaired exercise capacity in cardiovascular contexts.^11^^,^^25^^,^^28^^,^^29^ The present work lies in applying body composition phenotyping specifically to treatment selection rather than prognosis alone, extending concepts from oncology,^21^, ^22^, ^23^ heart failure,^24^ and aging populations^19^^,^^20^ into the peripheral arterial disease rehabilitation context.

Several important limitations warrant acknowledgement. The small sample size of eight patients limits the statistical power, generalizability, and precision of these effect estimates. The single-center design may produce results specific to our population that may not apply broadly. The relatively short 3-month follow-up period cannot assess long-term outcomes or the durability of conservative management. Missing data for some assessments reduces analytic precision. In particular, baseline CT body composition data were available in only two of the three patients requiring revascularization, meaning that the observed quantity-quality paradox between outcome groups is based on an extremely small subgroup and should be interpreted with corresponding caution. The correlational nature of our analysis cannot establish causation, only associations requiring mechanistic validation.^19^^,^^26^^,^^27^ Revascularization decisions were based on clinical judgment rather than strict protocol, introducing potential subjectivity.^1^ We segmented the cross-sections using AutoMATiCA, an automated body analysis framework based on neural networks, to build a segmentation map based on L3 CT scans without further editing needed.^39^ Different neural networks can make different segmentation maps. The absence of external validation means these exploratory findings require independent confirmation before clinical implementation. Finally, the value of this approach is contingent on CTA being obtained before the physiotherapy decision, which reflects tertiary center practice but may not be generalizable to settings where a noninvasive workup alone precedes conservative management. These limitations are appropriate for pilot feasibility work and distinguish what this level of evidence can conclude regarding technical feasibility from what it cannot conclude regarding predictive validity.

Methodological strengths include a prospective design with a standardized, pragmatic intervention reflecting real-world practice. The comprehensive assessment battery combined multiple body composition parameters with diverse functional measures. Automated CT analysis enhances reproducibility and reduces operator dependency.^16^^,^^17^ The real-world patient population with a high comorbidity burden strengthens generalizability to typical claudication cohorts.^37^ Complete outcome ascertainment ensured all patients reached treatment end points, and transparent reporting included nonresponders, missing data, and negative findings. Most important, the successful demonstration of technical feasibility in routine clinical settings establishes proof-of-concept for this innovative evaluation approach.

Future work should prioritize multicenter validation in adequately powered cohorts of 50 to 100 patients to confirm observed correlations and refine predictive models.^1^^,^^7^ The gold standard would be a randomized trial comparing standard approach with phenotype-guided treatment selection,^1^^,^^31^ evaluating both clinical outcomes and cost effectiveness. Mechanistic studies could elucidate why visceral adiposity impairs rehabilitation capacity and whether metabolic optimization improves outcomes in high-risk phenotypes.^26^^,^^27^^,^^34^ The development of automated algorithms with defined cut-points would facilitate clinical decision support.^16^^,^^18^ Integration with inflammatory and metabolic biomarkers might enhance predictive models.^11^^,^^19^

## Conclusions

This feasibility study establishes that CT muscle phenotyping represents a clinically actionable innovation for peripheral arterial disease physiotherapy selection. Although the small sample size mandates cautious interpretation of specific correlations, the successful demonstration of technical feasibility, combined with biologically plausible exploratory observations suggesting that muscle quality may prove more relevant than quantity and visceral adiposity may represent a dominant metabolic barrier, provides sufficient rationale for larger validation studies. If confirmed in adequately powered cohorts, automated CT phenotyping could be seamlessly integrated into existing diagnostic workflows, providing objective metabolic risk stratification to optimize treatment selection and resource allocation in conservative claudication management.

## Declaration of generative AI and AI-assisted technologies in the writing Process

During the preparation of this work the authors used Claude Sonnet 4 (Anthropic) to assist with manuscript writing and editing. The authors reviewed and edited the content as needed and take full responsibility for the content of the publication.

## Author Contributions

Conception and design: MP, OJ

Analysis and interpretation: MP, MK

Data collection: BR, LB, JD, MK

Writing the article: MP, OJ

Critical revision of the article: MP, BR, LB, JD, MK, OJ

Final approval of the article: MP, BR, LB, JD, MK, OJ

Statistical analysis: MP

Obtained funding: MP, MK

Overall responsibility: MP

## Funding

This study was supported by a Specific University Research Grant MUNI/A/1684/2025 provided by the Ministry of Education, Youth, and Sports of the Czech Republic and Internal Grant INT2023001 from Hospital AGEL.

## Disclosures

None.
